# Identifying barriers and potential solutions to improve equitable access to community eye services: an exploratory sequential mixed methods study protocol

**DOI:** 10.1136/bmjopen-2023-082975

**Published:** 2025-01-23

**Authors:** Luke Nelson Allen, Sarah Karanja, Michael Gichangi, Sailesh Mishra, Shalinder Sabherwal, Keneilwe Motlhatlhedi, Oathokwa Nkomazana, David Macleod, Min Kim, Jacqueline Ramke, Bakgaki Ratshaa, Malebogo Tlhajoane, Ari Ho-Foster, John Tlhakanelo, Nigel Bolster, Abhishek Roshan, Mohd Javed, Matthew J Burton, Andrew Bastawrous

**Affiliations:** 1 Clinical Research, London School of Hygiene & Tropical Medicine, London, UK; 2 Global Primary Care, University of Oxford, Oxford, UK; 3 Population Health and Primary Care, Kenya Medical Research Institute, Nairobi, Nairobi County, Kenya; 4 Ministry of Public Health and Sanitation, Division of Preventive Ophthalmic Services, Nairobi, Kenya; 5 Nepal Netra Jyoti Sangh, Kathmandu, Nepal; 6 Dr Shroff's Charity Eye Hospital Delhi, New Delhi, India; 7 Department of Family Medicine and Public Health, Faculty of Medicine, University of Botswana, Gaborone, Botswana; 8 Department of Family Medicine and Public Health, Faculty of Medicine, University of Botswana, Gaborone, Gaborone, Botswana; 9 London School of Hygiene & Tropical Medicine, London, UK; 10 Peek Vision, Berkhamsted, UK; 11 Sagarmatha Choudhary Eye Hospital, Lahan, Nepal; 12 London School of Hygiene and Tropical Medicine, London, UK; 13 Peek Vision, Berkhamsted, London, UK

**Keywords:** health equity, health services, health services accessibility, change management, international health services, primary health care

## Abstract

**Abstract:**

**Introduction:**

Access to care varies by sociodemographic group, with some groups facing higher barriers to care than others. This study will use novel methods to explore barriers and potential solutions as perceived by members of the population groups who are least able to access care. We aim to use rapid yet robust mixed methods that allow us to identify generalisable findings within each programme and testable service modifications to improve equitable access to care; delivering non-tokenistic findings within a matter of weeks.

**Methods and analysis:**

This is a multiphased exploratory sequential mixed methods study. We will use the same approach in four different screening programmes, in Botswana, India, Kenya and Nepal. First, we will conduct interviews with people purposively selected from the sociodemographic subgroups with the lowest odds of accessing care within each programme. We will explore their perceptions of barriers and potential service modifications that could boost attendance at eye clinics among people from these ‘left-behind’ groups. We will use a deductive analytic matrix to facilitate the rapid analysis of qualitative data. Space will be made for the inductive identification of themes that are not necessarily captured in the framework. Sample size will be determined by thematic saturation. Next, we will conduct a survey with a representative sample of non-attenders from the same left-behind groups, asking them to rank each suggested service modification by likely impact. Finally, we will convene a multistakeholder workshop to assess each service modification based on ranking, likely impact, feasibility, cost and potential risks. The most promising service modifications will be implemented and evaluated in a follow-on randomised controlled trial, the methods for which will be reported elsewhere.

**Ethics and dissemination:**

This project has been approved by independent research ethics committees in Botswana, Kenya, India, Nepal and the UK. We will disseminate our findings through local community advisory boards, national eye screening meetings, in peer-reviewed journals and at conferences.

**STRENGTHS AND LIMITATIONS OF THIS STUDY**

- We have developed a rapid qualitative approach that is designed to deliver rich and robust data with speed and relatively low costs.
- By using mixed methods, we are able to move from rich data to statistically generalisable findings that can be implemented across individual programmes.
- Our project is embedded within real-world programmes and will deliver actionable intelligence directly to policymakers, programme funders and programme implementers.
- Our proposed methodology places the experience and perspectives of ‘left-behind’ groups at the very centre of programmatic quality improvement.
- Our approach is based on a scoping review of rapid methods, however it is yet to be tested and validated.

## Introduction

### Equitable access to care

 Universal health coverage (UHC) has been described as the core of the health-related Sustainable Development Goals.^1 2^ As such, boosting access to community-based services has become an important global health priority.^3 4^ Inequitable access is a ubiquitous problem, with minoritised groups often facing the highest barriers to care.^5 6^ The ‘central transformative promise’ of the Sustainable Development Goals is to ‘leave no one behind and reach the furthest behind first’.^7^ Acknowledging that different groups face different barriers in different settings, WHO encourages health programme managers to routinely perform equity analyses in order to identify which specific groups are being left behind in each setting.^8^

Our group has produced guidance on how to perform these equity analyses using case studies from the field of eye care.^9^ Studies from Nigeria, Sri Lanka, Kenya and work in press from India, Botswana and Nepal show that different groups are being left behind by different programmes, ranging from elderly female widows in Sri Lanka to young men in full-time employment in Kenya.^10 11^ Once these groups are identified, the next step is to identify the unique barriers they face and potential solutions to improve access to care. Ideally, this work would be conducted quickly enough to inform programmatic decision-making.

#### Whose perspective do we want to hear?

Across all health service research, efforts to understand and address barriers to access have disproportionately focused on eliciting the opinions and perspectives of ‘experts’ and service providers at the expense of affected people and communities.^12^ Grounding elicitation work in the experiences and perceptions of service users and non-attenders is important both for ethical reasons^12 13^ and because their perceptions often differ from those of service providers.^14 15^ While elicitation studies from the field of eye care have largely been alive to this fact, there are still major issues: the approaches used to explore people’s perceptions have been disproportionately based on the use of closed questions and surveys, or on undertheorised and poorly described qualitative methods.^14^,^20^

#### Quantitative versus qualitative approaches for exploring barriers and solutions

The literature on barriers to accessing eye care is dominated by findings from in-person surveys that have been bolted onto population-based screening studies. These commonly take the form of a single survey item where participants are asked to choose or rank reasons for non-attendance from a preselected list of options.^17^,^1919 21^ This is also the approach used in *Rapid Assessment of Avoidable Blindness* surveys—of which >300 have been conducted in >80 countries.^27^ In our review of the literature, we only found two studies that provided a rationale for the list of barriers that they present to participants: Marmamula *et al* asked participants in South India to rank 15 barriers that had been generated by previous focus group work.^28^ However, none of the focus group participants were intended service beneficiaries or people with lived experience of trying to access eye care (all were service providers, public health experts and researchers).^29^ Furthermore, while the people responding to the final survey all had some form of vision impairment, they had not necessarily ever been referred to a service, which may explain why ‘lack of felt need’ and ‘lack of awareness’ were the most frequently selected barriers. Sengo *et al* performed a literature review and interviewed 25 people in Mozambique with vision impairment to identify which barriers should be used in a wider survey.^30^ However, the exercise was inadequately described, and the authors do not provide any detail on how the qualitative data were analysed.

Almost all surveys use a familiar list of barriers that commonly recur in qualitative studies, including high costs, long distances, transport issues, low trust in service providers, perceptions of low service quality, communication challenges, fear, scheduling issues, low awareness of services on offer, lack of a chaperone and the perception that vision impairment is not a significant impediment to function.^14^,^1820 22^ The main limitation in using surveys with these preselected items is that other important factors may be at play in a given population, but it is impossible to ascertain what they are without using open questions.^33^ Methods to elicit these barriers do not have to be particularly sophisticated: even though Sengo *et al* appear to have used relatively basic qualitative methods, their study still uncovered novel and important barriers including overcrowding in the local hospital, self-medication and the use of spectacles bought on the street.^30^ Similarly, while the method outlined by Marmamula *et al* to interview 199 elderly non-attenders provided no reference to theory, no underlying framework and no detail on the analytical approach, the work proved vital to understanding local contextual issues, with two-thirds of respondents citing novel barriers including lack of family consent and the adverse impact of other health conditions.^34^ These factors would not have been elicited from participants through a standard survey.

When are *any data* better than *no data*? Poorly designed qualitative studies can lead researchers to the wrong and sometimes harmful conclusions, just as flying blind without any understanding of the issues faced by service users can lead managers to introduce well-meaning ‘improvements’ that carry negative unintended consequences. We would argue that using appropriate, theory-driven qualitative methods with a sensible sample and well-described methods is actually a very low threshold to clear and can add real value at low cost in settings where the alternative is not using any open questions at all.

#### Previous qualitative studies that have examined access to eye care

There is a paucity of studies that have used qualitative methods to explore barriers to care. Ahmad *et al* used an open-ended survey question and content analysis to identify barriers to accessing eye care among the general population in Karachi. Unsurprisingly, given the population included, low perceived need was a major reason for not seeking care, however issues around health beliefs and cultural attitudes were surfaced that represent important issues for local health teams to engage with.^35^ Zabeck *et al* used structured telephone interviews to explore barriers to access among 28 Americans who had become blind. Using a constant comparative approach they found that social support structures and personal readiness to change were important factors for some people, alongside familiar themes of geographic access and low trust in providers.^36^ Elam and Lee conducted content analysis on data from four focus groups with American community members at risk of not attending eye services. Issues around health insurance, racism, unfriendly service at the clinic and procrastination supplemented familiar themes of cost, trust and fear.^31^ Kulkarni *et al* conducted in-person interviews with transgender people and sex workers with vision impairment in Pune, India, followed by focus group discussions with service providers. Their interview topic guide used deductive (ie, pre-identified) themes to structure the questions, and made space ‘to identify previously unexplored domains’. It appears that the provider focus groups were conducted in parallel in order to triangulate findings from the interviews. This approach was also used in studies led by Owsley *et al* and Okoye *et al*; both triangulated interview data from the target population with the perspectives of service providers, and Okoye *et al* also engaged with policymakers.^14 15^

#### Which population should be sampled?

While most eye care studies that assess access have sampled participants from either the general population or the population of intended service beneficiaries, three studies have specifically engaged ‘non-attenders’ (we note that this term is not perfect as it implicitly places responsibility for access onto users rather than services). It is likely that those who have been diagnosed with an eye condition; referred and not managed to access those services will have greater insight on the barriers to access and potential solutions than members of the general population who do not have this lived experience. Chou *et al* used a survey with preselected items to elicit reasons for non-attendance,^20^ but Gower *et al* used semi-structured telephone interviews which enabled participants to cite barriers that the researchers might not have previously considered.^16^ Similarly, Marmamula *et al* used in-person semi-structured interviews to elicit reasons for low eye clinic access among elderly care home residents.^34^

#### Theory

Very few of the qualitative studies that we found grounded their analyses in theory or a conceptual framework. While there are many different conceptual frameworks on generic barriers to accessing services,^37^,^39^ we are not aware of any that have been developed for eye care beyond the Australia-focused tripartite division of ‘predisposing’, ‘enabling’ and ‘need’ characteristics described by Keefe *et al*.^40^ Despite the breadth of eye service utilisation studies that have been conducted in the past two decades, it seems that it is rare for quantitative or qualitative eye care studies to use theory to inform the design of data collection activities or guide interpretation of findings. Positively, unlike healthcare access research from other fields, approaches that are grounded in eliciting the views of people and communities (as opposed to ‘experts’) are the norm, but these disproportionately sample form the general target population, rather than those with lived experience of being unable to access care.

## Study aim

In this study, we aim to develop a rapid, theory-based, scientifically robust approach that can be used to elicit barriers to accessing care and potential solutions through engagement with ‘non-attenders’ from sociodemographic groups that experience the lowest overall access rates when referred from screening programmes. We intend to test this approach in four different eye screening programmes running in Botswana, India, Kenya and Nepal and then apply the findings within the same services with the ultimate aim of improving equitable access to care. Findings from one programme will not be applied to the others, although learning will be shared across sites.

## Objectives

1. In each individual screening programme, conduct interviews with people from the relevant left-behind groups who have not been able to access clinics (identified in previous equity analyses) to explore barriers and potential solutions.
2. In each individual screening programme, conduct phone interviews with a representative sample of people from the relevant left-behind groups, asking them to rank each of the mooted solutions.
3. In each individual screening programme, convene the programme funder, programme implementing team, community representatives and national eye care policymakers at a workshop to review the ranked solutions and select one or more for implementation and evaluation.

### Programme-specific requirements

The nature of the screening programmes imposes a methodologically challenging set of requirements. Given that some programmes screen entire regions in a matter of months, the approach that we use must be able to deliver service modifications rapidly enough to benefit a reasonable proportion of the remaining intended beneficiaries; ideally within 1–2 months. Next, rather than presenting participants with a preselected list of barriers and service modifications and then asking them which are most important, we want to use open questions that allow participants to use their own words to identify issues and approaches that the research team may not have necessarily considered. We recognise that coding and interpreting these responses requires time—however speed is a key objective to ensure feasibility when running at large scale on tight resources. Peek Vision - the social enterprise that provides the screening programme software - is keen for its programme partners to use any resultant methods that can improve referral uptake, but the cost of these research activities will ultimately be borne by programme funders and will likely be offset by a reduction in the total number of people screened. As such, there is considerable pressure to keep the overall costs as low as possible. A related constraint is that local teams will only have access to a small number of staff with basic research training. We note that the availability of experienced qualitative and mixed-methods health system researcher staff is low across many low- and middle-income countries.^41 42^ Next, as stated above, we want to base decisions on the experiences and perspective of those directly affected: people who have been identified with an eye need and referred, but who have not been able to access services. Furthermore, we aim to focus on the needs of the sociodemographic group with the worst access to care (‘reaching the furthest behind first’) so that any improvements disproportionately benefit these groups, thereby improving equity, in line with the principle of proportional universalism.^6 43^ Finally, despite being rapid, inexpensive, non-prescriptive, equitable and primarily conducted by non-experts, we are committed to using robust methods to deliver valid, non-tokenistic findings. This is vital in order to inform programmatic changes that stand a chance of meaningfully improving access rates (box 1).

Box 1 **Our improbable wish list**

We want to develop a rapid elicitation tool that can:

- be largely conducted by non-experts, although with expert supervision;
- deliver a set of barriers and potential solutions within weeks-months, rather than months-years;
- use open questions rather than relying solely on a predefined list of response options;
- provide barriers and potential solutions that are generalisable;
- gather data from non-attenders from sociodemographic groups with the lowest attendance rates within each programme;
- be conducted relatively inexpensively;
- and is methodologically robust.

## Approach

### Philosophical paradigm

Our aim requires methods that span the space between constructivist and positivist philosophical paradigms.^44^ While the task of seeking to understand perceptions of barriers and solutions is primarily phenomenological, we intend to generalise the findings (ie, make statistical inferences) and develop service modifications that will be applied across entire programmes within each country. To traverse this philosophical rift, we will use a pragmatist paradigm, originally advanced by Charles Sanders Peirce.^45 46^ Pragmatism holds that ‘truth’ is determined by practical application and consequences, and it is agnostic on the type of research techniques used as long as they answer the research question.^45 47^

### Undergirding theory

**Figure 1:**
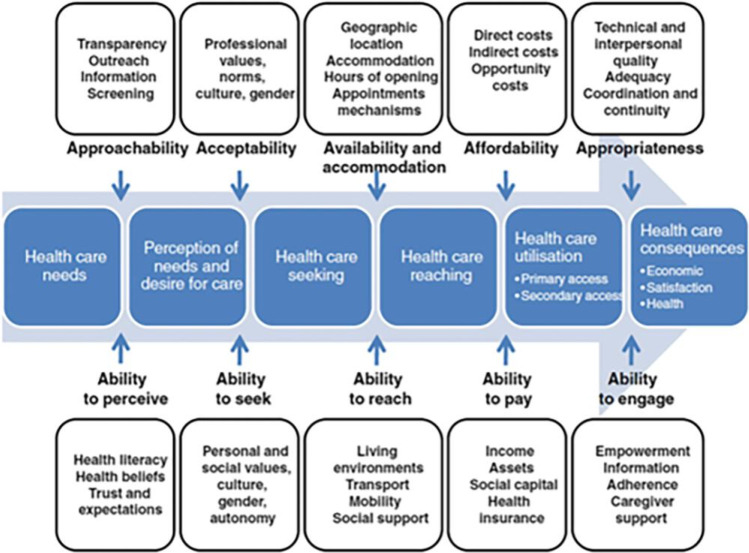
The framework by Levesque *et al*.

There are a large number of conceptual frameworks on access to health services.^3739 48^,^52^ As our ultimate aim is to elicit ideas for ways of improving services to boost equitable access, we have elected to use the popular model developed by Levesque *et al* (figure 1)^39^ that divides factors into those pertaining to services and those related to potential service users. We want to focus our analysis on areas that we are most able to change, that is, the structure, staffing, organisation and communications of eye services, in contrast to user characteristics like social support networks, assets and health literacy, which are important but much harder for programme managers to influence.

The framework by Levesque *et al* is based on the findings of a systematic review that identified five determinants of healthcare access: approachability, acceptability, availability and accommodation, affordability and appropriateness, along with corresponding abilities to perceive, seek, reach, pay for and engage with services. These factors feed into a process of seeking care that resonates with the Tanahashi framework^53^ and the concept of effective coverage,^54^ that is, access is predicated on a series of steps that include perceiving an initial need, desiring care, seeking out potential providers, travelling to the location at a time that it is open and staffed, and having sufficient resources to be seen. Access only occurs when the requisite supply and demand side elements are in place.

Obrist *et al* have developed an aligned model with a specific emphasis on ‘analysis for action’ and application in low-income settings.^37^ Their five dimensions—availability, geographic/logistical accessibility, affordability, adequacy and acceptability—overlap with those presented by Levesque *et al*, and are supplemented by five types of livelihood assets that determine ability to recognise need and seek out health services: human capital (local knowledge, education, skills); social capital (social networks and affiliations); natural capital (land, water and livestock); physical capital (infrastructure, equipment, means of transport) and financial capital (cash and credit). The authors note that many of these assets are influenced by macroeconomic and political conditions, climate change and many other forces over which people have very little control, and are also difficult for service managers to influence directly.^37^

### Methodology

We require mixed methods that draw on the strengths of both qualitative and quantitative approaches to answer a multilayered question: what are the main barriers to accessing eye services in each location (ie, each individual screening programme in each of the four countries) and what can be done about them?

Qualitive methods deliver rich, descriptive data based on interviews, discussions and/or observations with a select number of participants who are often purposively chosen because of their specific characteristics. As such, the findings can be transferred to similar cases and contexts, but they are not intended to be generalisable. In contrast, quantitative methods deliver numerical data and—with representative sampling—are able to provide evidence for causality, generalisability and magnitude of effect.^55 56^

We will use an exploratory sequential mixed methods approach; starting with qualitative methods to explore non-attenders’ perceptions of the barriers and potential solutions in each setting. We will use the identified themes to develop a unique, user-derived list of potential service modifications within each screening programme. We will then use quantitative methods—a survey—to establish which of these are perceived to be the most impactful through engagement with a representative sample of non-attenders, effectively validating or ‘sense-checking’ the qualitative findings with a larger, representative group. The ranked suggestions for service improvements will then be taken to a multistakeholder workshop where the top-ranked solutions will be considered for implementation based on their likely impact, feasibility, cost and potential risks.

### Context

Our research team is studying access to eye services in screening programmes that use Peek Vision app-based screening and referral management systems in Botswana, India, Kenya and Nepal.^57^ These large screening programmes are identifying hundreds of thousands of children and adults who need glasses, cataract surgery and other cost-effective, life-changing interventions. We selected these four countries because they represented different populations and screening programme types; had screening programme commencement dates that aligned with our project timeline and were overseen by managers with pre-existing academic collaborative relationships with the London School of Hygiene & Tropical Medicine (LSHTM) team.

**Figure 2:**
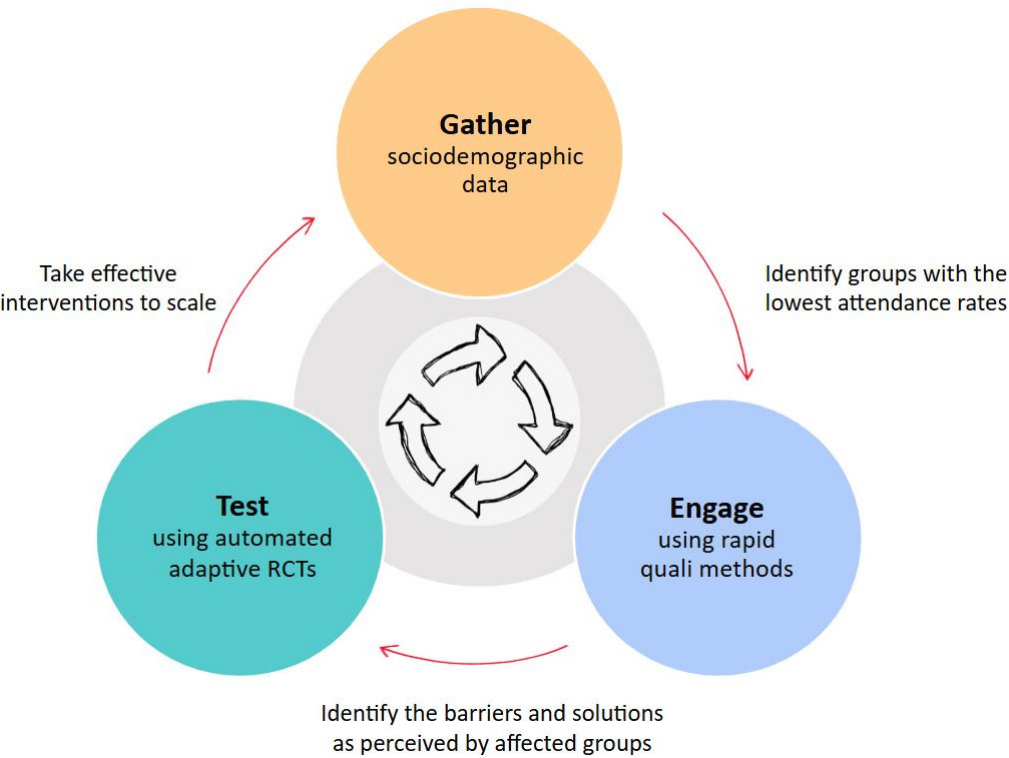
This current project represents the ‘Engage’ component in the wider ‘IM-SEEN’ continuous improvement project. RCT, randomised controlled trial.

This project constitutes the ‘Engage’ element of the broader ‘IM-SEEN’ continuous improvement approach.^58^ It is preceded by activity to gather sociodemographic data from those being screened in each setting and the identification of which groups experience the lowest access rates (figure 2). The purpose of the current ‘Engage’ project is to gather and prioritise a list of barriers and potential solutions, grounded in the perceptions of left-behind groups. A follow-on project will use a randomised controlled trial (RCT) to test whether the most promising solution(s) actually equitably improve access to services.

## Methods and analysis

### Overview

**Figure 3:**
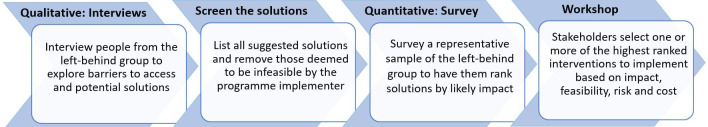
Overview of the sequential mixed methods approach.

We will use a four-stage rapid exploratory sequential study design (figure 3). First, we will conduct telephone interviews with non-attenders purposively selected from the sociodemographic subgroup that has the lowest overall access rate within each screening programme. We will explore their perceptions of barriers, potential solutions and compile a long list of all suggested solutions/service modifications. We will discuss the long list with the programme funder and implementer to rule out any suggestions that are felt to be completely unfeasible, for example, providing helicopter transport for everyone who is referred. All decisions to remove suggested interventions will be independently reviewed by the director of Peek Vision. Next, we will conduct a telephone survey, asking a representative sample of non-attenders from the same left behind group to rank the remaining suggestions by likely impact. Finally, this list of prioritised service modifications will be put to a group of programme funders, programme implementers, community representatives and eye care policymakers. Participants will review the top-ranked service modifications and select one or more to test based on likely impact, feasibility, cost and potential risks. The intervention that is perceived to offer the best value according to these criteria will be implemented and evaluated within the context of an embedded pragmatic RCT, the methods for which will be reported elsewhere. This approach will be conducted independently in screening programme, that is, potential solutions identified in Uttar Pradesh will not be used to inform service modifications in any other setting. Figure 3 provides an overview of the study elements.

### Developing a rapid qualitative approach

Our study is not the first that seeks to use rapid and low-cost qualitative methods that can be led by less-experienced researchers (early career researchers and those with basic rather than postgraduate training) to answer an open question. Rapid methods have been in use for over 30 years, as described by Beebe,^59^,^61^ Handwerker,^62^ Pearson,^63^ Bentley *et al*,^64^ Haenel *et al*^65^ and Johnson and Vindrola-Padros.^66 67^ There are also examples of rapid qualitative studies that have intentionally used teams of less experienced researchers.^68^

Rapid qualitative methods are often used to reduce time and costs, and to improve efficiency, accuracy and ‘obtain a closer approximation to the narrated realities of research participants’.^67^ These studies generally take between a few days to a few months to complete depending on the design, with most taking a couple of weeks.^66 69^ A large number of dedicated approaches have been developed, including ‘rapid ethnographic assessment’,^70^ ‘participatory rural appraisal’,^71^ ‘rapid rural appraisal’,^72^ ‘rapid appraisal’ (a form of ‘rapid qualitative enquiry’),^59^ ‘rapid assessment procedures’^59 65^ and ‘rapid assessment response and evaluation’.^73 74^

In their review of rapid qualitative methods, McNall and Foster-Fishman identified the following key features: these studies commonly use mixed and multimethod approaches to triangulate data; they tend to be participatory, with representatives of the target population involved in planning and implementation; they are team-based, with all members working collaboratively on all aspects of the research process and they are iterative, with data being analysed as they are collected and early findings being used to guide additional data collection until theoretical saturation is reached.^75^ The authors also note that the central trade-off is between speed and trustworthiness. Vindrola-Padros and Vindrola-Padros identified several key challenges that apply to all rapid qualitative approaches, as summarised in table 1.^69^

**Table 1:** Risks of rapid research, as described by Vindrola-Padros and Vindrola-Padros^69^

Design feature	Potential risks
Sample size and representativeness	‘Dependency on most accessible informants and loss of multiplicity of voices’.
Community participation	‘Local research assistants are not always available, have the required skills or willingness to take part. Training takes time. Research undertaken by researchers without an anthropological background might limit the quality of the study’.
Team-based approach to design, data collection and analysis	‘Recruitment might be an issue and clear roles in the field need to be outlined’.
Brief engagement time	‘Inability to capture changes over time, understand all relevant social and cultural factors at stake, or conflict and contradictions… New researchers might get more attention, but lack familiarity with the study area. Prolonged engagement often increases credibility and internal validity. Prolonged engagement might also lead to stronger relationships between research participants and the field researchers. The rapid study timeframes might not allow researchers to critically analyse the position they play in the field site and their role in the collection and analysis of data’.
Governance	‘Time pressures should not deter researchers from undergoing the required governance and informed consent processes’.

Many of these risks can be met head-on, for example, by obtaining ethical approval and informed consent, thinking carefully about team roles and purposively sampling from the most minoritised groups. The extent to which community members can or should be engaged is dependent on the study aims and local contextual factors. The greatest challenges are around developing robust findings based on a brief engagement period. Triangulation can help (ie, using multiple methods or data sources to develop a comprehensive understanding of phenomena^76^ but this limitation renders rapid methods unsuitable for qualitative research projects that require a deep, emic understanding of complex phenomena and issues.

Building on established rapid qualitative analysis, our team has conducted a scoping review to identify rapid approaches that have been specifically used to assess barriers and solutions to improve access to community health services.^77^ We identified a number of innovative methodological techniques that can be used to minimise the length of time between data collection and implementation of the final set of findings. Many of these design features are best suited for deductive framework analyses where participants’ experiences are sought in relation to a clearly defined a priori research question. In our case, the question is: “what stopped you attending and what could be done about it?”

In line with findings from a broader review of rapid methods,^67^ we found that many approaches focused on eliminating or expediting the transcription phase, either by performing simultaneous data collection and analysis, or by coding data directly from audio. This is a common design feature of studies that use ‘RAP’ sheets (*Rapid Assessment Procedure* data templates): data collectors enter quotes and/or open codes into analytic matrices during the interview or afterwards, working directly from the audio recording.^68^ Clearly, this limits the depth and richness of the analysis, making the approach inappropriate for complex and nuanced qualitative research questions. However, many applied research teams have used contemporaneous analysis to elicit meaningful and non-tokenistic findings in contexts where there is a narrow and clearly articulated question. The few studies that have compared these direct coding approaches with coding based on transcripts of the same interviews or focus group discussions found that both approaches generated similar themes with acceptable reliability.^78 79^

In our scoping review, we found that the most commonly used application of direct coding was in entering data into a deductive template during the interview and/or directly afterwards, working from handwritten notes and/or the audio recording rather than a transcript. The loss to analytical power from obviating a written record can be partly offset by having data collectors co-located, which has been shown to lead to informal discussion and analysis through natural debriefing conversations.^68^ Some researchers have formalised this process, holding group meetings directly after data collection to collaboratively summarise, analyse and interpret findings, such as in the work led by Jalloh *et al*.^80^

Many rapid studies seeking to understand barriers to healthcare access make use of deductive templates or matrices to chart data or use ‘one sheet of paper’ techniques to aid rapid analysis and presentation of findings.^80^,^84^ Miles and Huberman have argued that data reduction, display and the drawing of conclusions happens simultaneously in qualitative analysis,^85^ and that the use of matrices can drive credibility and trustworthiness.^86^ While the use of a priori codes and/or themes to populate a framework template may save time at the analysis stage and potentially reduce the skill requirement, the burden of work is shifted to an earlier stage of the project rather than eliminated. A further issue is that deductive approaches are misaligned with the general aim of moving away from preselected checklists of potential barriers and making space for affected people to describe the issues in their own words, ideally surprising researchers by describing barriers and potential solutions that had not previously been considered and by ‘making the familiar strange’.^87^ However, Pope and Mays argue that virtually all qualitative analytic approaches involve a combination of inductive and deductive reasoning, and the use of a deductive framework does not necessarily preclude inductive coding.^44^ They make a strong case for ‘abductive’ reasoning that benefits from the efficiencies of the deductive framework approach while ‘leaving space for more inductive identification of themes and issues not predicted at the outset’.^44^

Based on the lessons learnt from reviewing the literature, we aim to adopt several rapid techniques to increase the speed and affordability of our qualitative research element, detailed below.

### Interviews with non-attenders or their proxies

#### Recruitment and sampling

Participants in Peek-powered screening programmes operating in Botswana, Inda, Kenya and Nepal provide their name, a contact number and—if they consent—data on approximately 10 sociodemographic domains including age, sex, education, income, assets and health status (the unique lists for each national programme and selection processes have been detailed in a previous IM-SEEN publication).^88^ Peek has consent procedures and agreements that enable these data to be shared with our embedded research team. In each country, we will conduct quantitative equity analyses to identify which sociodemographic characteristics are most strongly associated with non-attendance in each programme. This work has already been completed in Meru, Kenya, where we found that younger people, males and those working in sales, services and manual jobs were the least able to access care. In our intersectional analysis, we found that only 14% of young men who worked in sales, services and manual jobs accessed clinics in comparison with 46% across the entire referred population.^89^

In line with the global health principles of equity and health for all, we will purposively engage with the sociodemographic groups in each setting that experience the lowest access rates. We will purposively recruit people who have been referred but have not accessed care within 2 weeks of their appointed date from the left-behind subpopulation.

We will have the phone numbers for every person who did not access care from the left-behind subpopulation. We will generate a spreadsheet that contains each person’s name, unique study ID number, phone number and screening date. The preceding equity analysis will have given us the characteristics most strongly associated with non-attendance in each programme (eg, low-income Muslim agricultural workers). While everyone on the call list will belong to this left-behind group, other characteristics (eg, age, sex, income, education, location, etc) will vary. We will ensure that we interview people with a broad spread of the characteristics not found to be strongly associated with non-attendance in order to ensure that we hear a wider range of voices. We will order the names randomly, using a random number generation function in R or Excel, and then work down from the top of the list to start the interviews. When we identify the characteristics that have not yet been represented, we will scroll down and call the first person on the randomly sorted list who has the characteristics we are keen to include.

Our sample size will be determined by the point at which we reach thematic saturation. Empirical evidence suggests that the majority of all themes and concepts emerges within the first 5–6 interviews^90 91^ and that saturation is usually reached within 9–17 interviews when conducted among a relatively homogeneous population.^92 93^ We will use the approach by Guest *et al* to assessing saturation, using a prespecified base size (ie, a minimum number) of 12 interviews, followed by runs of two interviews and a 0% new information threshold. In other words, we will stop conducting new interviews once no new themes emerge after two interviews in a row, with a minimum sample size of 14. We will budget conservatively for 20 interviews in each location.

#### Data collection

Small teams of data collectors will conduct interviews in each country. All data collectors will have at least basic training in qualitative methods but will not necessarily be full-time qualitative researchers. Where possible we will recruit, and train lay members from the target population to assist with data collection. All data collectors will be fluent in the language(s) spoken by the target population.

We will use semi-structured telephone interviews, directly exploring participants’ views of the issues that prevented them from attending clinic and the potential service modifications that they feel would have enabled them to attend. We will call potential participants and explain the study, and then seek recorded audio consent. All interviews will be conducted in the participant’s own language. Data collectors will work in pairs. They will be trained by a senior bilingual qualitative researcher who will also perform daily debriefs during the data collection period.

While face-to-face interviews undoubtedly offer richer data in comparison with telephone interviews, we have opted for the latter on the basis of feasibility. Peek do not collect people’s home addresses, and even if we did have this information, the national screening programmes cover extremely large areas, meaning that it might take weeks of travel to conduct the interviews. In contrast, multiple phone interviews can be conducted each day, with much lower costs, while avoiding the personal safety risks to data collectors that come with extensive travel. A number of methods papers have argued that qualitative findings do not vary significantly between telephone and in-person modalities.^94 95^ Even so, we will conduct an embedded comparison study to assess the differences in data richness obtained from telephone versus face-to-face interviews.^96^

Data collectors will try to contact each interviewee three times, calling at different times of the day. If they are unable to make contact, they will move down the randomly sorted list and try the next non-attender. Interviews will be audio recorded. The recording will include the participants’ unique identifier, the consent process and—if given—confirmation of consent to participate. The following interview items will be used:

##### Barrier elicitation questions

- In your own words, can you talk me through why we didn’t see you/your child at that clinic?

##### Probing questions

- Are there any other factors that prevented you/him/her from attending?
- Is there anything else you’d like to share?

##### Solution elicitation questions

The last part of the interview is exploring whether there is anything we could do to address these barriers and make it more likely that other people like you/children like [child’s name] will attend in the future.

- So, to start, what would make the biggest difference?

##### Probing questions

- What else would help?
- What other changes could we make to the programme that would make it easier for you/children like [child’s name] or people like you/children like [child’s name] to attend?
- Are there any other specific changes that we could make to the way that the programme or eye clinics run?

### Qualitative analysis

One data collector will perform the semi-structured interview while the other listens in and takes notes on the times that main themes are mentioned. Immediately after the interview has concluded, the data collectors will listen back to the interview recording and navigate to the noted times. They will then type out the full quotes for each barrier or proposed solution verbatim into an analytic matrix, with one interviewee per column and one theme per row.

We have chosen to use this direct data entry approach because it is faster than generating and then working from transcripts, and because the nature of our (relatively simple) research question is more descriptive than explanatory. We have developed a bespoke deductive matrix that is grounded in the access models of Levesque *et al*^39^ and Obrist *et al*.^37^

### Development of the analytic matrix

**Figure 4:**
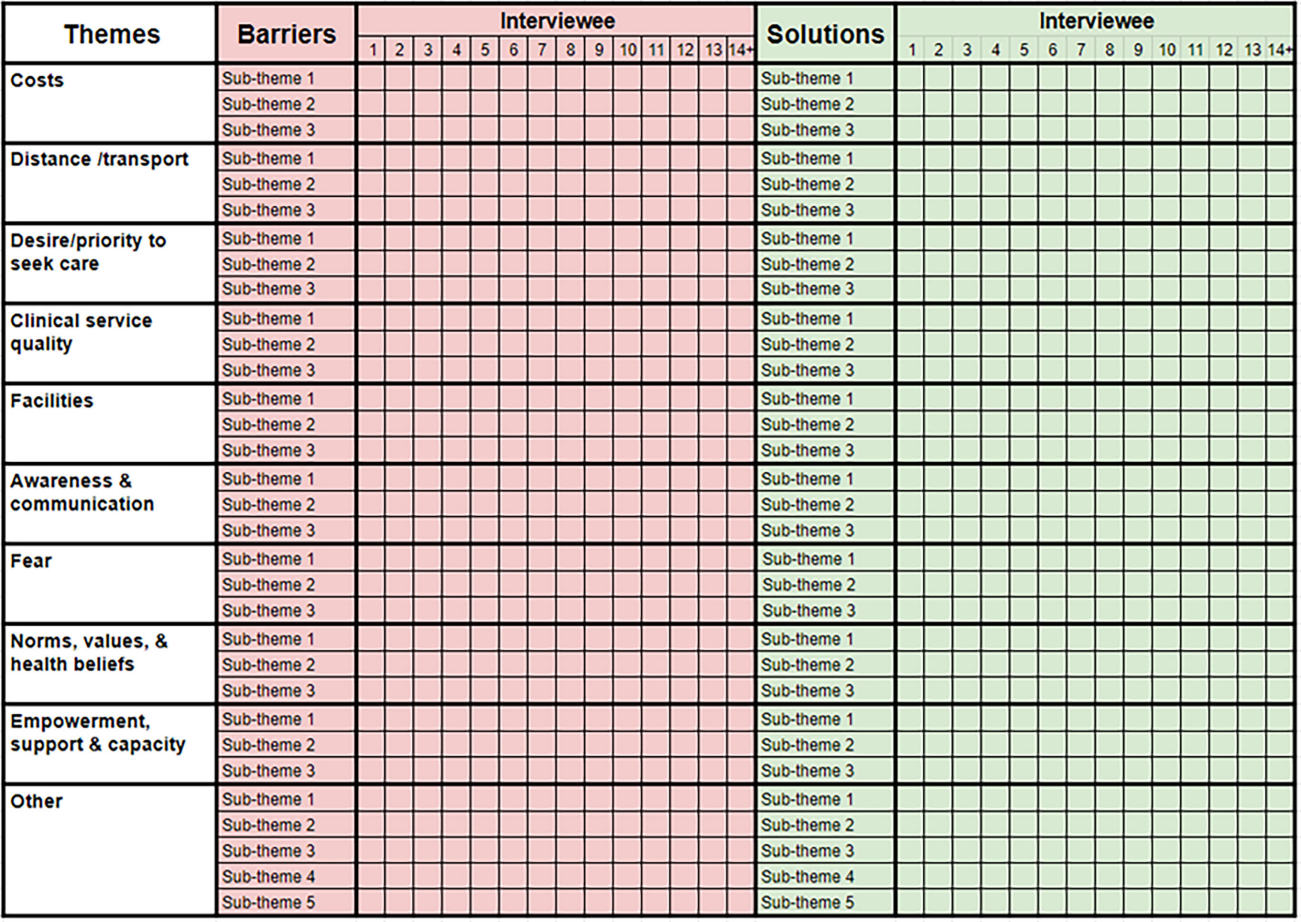
The analytic matrix.

We first mapped the Obrist dimensions to the service domains identified by Levesque *et al* (online supplemental table 1). Next, we selected domain descriptors that we felt captured the essence of each unique element from across the two frameworks (online supplemental table 2). We felt that ‘availability’ domain by Levesque *et al* straddled two different concepts: those related to distance/transport and facilities. Next, we added in the domains that pertain to users, mapping them to the service domains and providing a unified descriptor (online supplemental table 3). The framework by Levesque *et al* identified three areas that do not naturally correspond with service characteristics: themes around the desire to seek care, the capacity to participate in care (eg, though shared decision-making with a clinician or medication concordance) and empowerment and social support. Next, we mapped the common barriers that were identified in our literature review of the existing eye care literature to the unified descriptors of service and user domains (online supplemental table 4). Finally, we reconfigured this table to create a deductive template that can be used to enter quotes during and directly after each interview. The whole point of using interviews rather than a (much faster and less expensive) survey approach is to be able to uncover barriers and potential solutions that the research team had not previously considered. As such, the template, interview prompts and data collector training all emphasise the ‘other’ column. figure 4 illustrates our analytic matrix.

### Process for completing the matrix

During the interview, data collectors will expand the column width for the relevant interviewee number (eg, interviewee 1). They will type short quotes on each barrier into the relevant row, using the participant’s own words. Data collectors will repeat the process when asking for potential interventions that would have made it possible to attend, adding ideas to the matrix. They will probe for further forms of service modification (which we are able to change) that would make a tangible difference.

Directly after the interview, the data collectors will listen back to the audio recording of the interview and expand on and correct the quotes that they noted during the interview. All quotes will be directly translated into English. Data collectors will replace the ‘subtheme n’ text in the barriers and solutions columns with add their own (inductive) codes, for instance, ‘long queue at clinic’, ‘cost of spectacles’ or ‘rumours of inexperienced staff’. The number of subthemes is not limited; new rows can be added as required. As stated above, after a minimum of 14, interviews will continue until no new subthemes emerge from two successive interviews. Data collectors will debrief with experienced national qualitative research leads each day in person or online. The national leads and the international research manager will collaboratively check quality and consistency of data entry, review all quotes and subthemes and assess when thematic saturation has been reached. Once qualitative data analysis is complete, all audio recordings will be deleted.

### Use of findings

Once saturation is reached in a given programme, the research team will use the full matrix to generate a list of all the individual barriers and solutions that arose from the interviews. These may include things like sending SMS reminders, reducing the distance that people have to travel or altering the way that people are counselled before being referred.

The long list of solutions will be reviewed by the local programme funder and programme implementer to rule out any service modifications that are completely unfeasible—such as paying people US$100 to attend, or providing free individual transport for every participant. The short list of potential service modifications will form the basis of a survey that will be sent to a wider sample of non-attenders in order to identify the most promising actions at a generalisable level. Each programme will generate its own list of barriers and solutions.

### Survey

Once we have generated a list of potential solutions, we will perform a survey to rank these in terms of perceived impact. Again, this approach is programme specific. At no point are we applying findings from one programme to another. As stated above, we will have a complete list of every non-attender belonging to the sociodemographic group with the lowest overall attendance rate. We will administer a telephone survey to a representative sample of non-attenders from this group, excluding all of those who have already been interviewed. We will use a 95% CI, a 5% margin of error and a conservative assumption that the total population size is 1 million people (with the same characteristics as the most marginalised group). This renders a sample size of 384.

We will use computer-generated numbers to obtain a random sample of non-attenders to call. Data collectors will seek verbal audio recorded consent before reading through the full list of potential service modifications that arose from the interview stage. Respondents will be asked to rank each suggestion from 1 to 3 on a simple Likert scale:

1. It would make a big difference, that is, if we introduced this change then you or people like you would definitely attend.
2. It would make a moderate difference, that is, it would greatly increase the chances, but it would not be enough by itself to guarantee attendance by itself.
3. It might make a small difference, that is, it might help a few people, but the impact is likely to be minimal.

We will calculate the average score for each service modification and generate a ranked list. Workshop participants will review the ranked list and select the most promising service modification to implement and evaluate.

### Workshops

Our team already has formal agreements and pre-existing working relationships with Peek programme leads, programme funders, programme implementers, eye care policymakers and community advisory boards in each location. In each country, we will invite these stakeholders plus additional representatives from the left-behind group to a 90 min workshop to review the study findings and select one or more service modifications to implement. Workshop discussion will be led in English (the working language of the project in each country) by a facilitator from our research team. The lead researcher will present a brief overview of the barriers and potential solutions suggested by non-attenders and their proxies, and then lead a discussion to explore the groups’ perceptions of which barriers they can realistically address and which solutions offer the best balance of impact (based on survey respondent scores), cost, risk and feasibility. The aim is to identify promising service modifications that can be deployed and tested using RCTs to equitably improve access to care.

The process of decision-maker group discussion aligns with rapid methods that use group discussion with the ultimate research users as a key part of data analysis, interpretation and application. The workshop will close with the identification of the most promising service modification to test and discussion of next steps.

### Output

The primary output of this mixed methods study will be the selection of one or more feasible service modification(s) that has been identified by intended service users and agreed by service managers. This process will conclude during the workshops held in each country. The selected interventions will be tested across the relevant programmes using an adaptive randomised trial design, as part of the broader ‘IM-SEEN’ approach.

### Ethics and dissemination

#### Institutional review

We will seek ethical approval from the LSHTM ethics committee and all relevant ethics committees in Botswana, India, Kenya and Nepal.

#### Consent

##### Consent to be contacted for recruitment

In the screening stage that takes place before this project’s elicitation activities, written tick-box consent will be sought to use personal and contact data to recruit non-attenders for this current study. We will use the patient’s preferred language for the provision of patient information and consent. In each setting, the translations we use will be written by bilingual researchers, back-translated and the independently checked by second bilingual researcher. Participant information and consent wording will be read out loud in the presence of an independent witness for those who cannot read. Our team is fully embedded in the screening programmes in each country, and memoranda of understanding are in place to govern the sharing of data (including records of consent) between parties.

##### Consent wording used at screening

I understand that my/my child’s anonymised data may be shared with other researchers or online in a public repository for research. I understand that I may be contacted by Ministry of Health partner organisations inviting me to participate in future studies to improve access to eye care services. I understand that I can call [phone number] for free to ask any questions; that my decision will not affect the care that I/I or my child receives; and that I can change my mind at any time.

##### Consent for telephone interviews

For the qualitative interviews, we will call potential participants and provide information about the purpose and risks of the telephone interview using an appropriate version of the Botswana script shown below. Potential participants will have the opportunity to discuss the study and ask questions.

Hello, my name is___________. I am a researcher from the University of Botswana, working with the Ministries of Health and Basic Education on the Pono Yame eye screening programme.

Your child recently had their eyes screened at school and was found to need further assessment. Our records indicate that, like many other children, they were unable to attend that appointment.

You are being contacted because you have previously provided consent to be contacted by Ministry of Health partner organisations regarding research being conducted for eye care services. I am calling to invite you to participate in a 30-minute interview. Your participation is completely voluntary. This means that you do not have to do it unless you want to.

We want to understand the barriers that prevented your child from attending. We are also asking about how we could change the Pono Yame programme to make it easier for children to attend appointments.

Before agreeing, here is the background information that you need to know:

We have invited you because, like many other referred children, your child did not attend. We want to hear about the issues that you personally faced that prevented your child from attending, and your ideas on how to make things easier. In total we are aiming to interview about 20 people.

Who are we? I work with a group of researchers from the University of Botswana and the London School of Hygiene and Tropical Medicine. We are working to improve the national Pono Yame eye screening programme that will visit every school in the country. The leaders of the research are Prof Keneilwe Motlhatlhedi and Dr Luke Allen.

We will take the responses from all of the interviews and discuss the ideas for improvement with the leaders of the national programme. We hope to use your suggestions to make the programme work better.

We are also conducting a set of face-to-face interviews and online surveys with other parents and guardians. We want to compare the responses we get from these different approaches.

In this 30-minute interview there are no risks to you or your child. If you agree to take part, we will send you a 100 pula airtime voucher to compensate you for your time. It is important to note that agreeing or declining to take part does not have any impact on your child, their schooling, or the services they receive.

You can stop the interview at any time.

I will record the interview. Our team will anonymise your data and keep it safe and secure on a password-protected computer in London. When the study is completed, we will write-up our findings and publish them online so that other researchers can use the information to help people in other places.

The University of Botswana and London School of Hygiene and Tropical Medicine ethics committees have both approved this study.

You can ask me any questions you like now. I can also give you the email address and phone number of the lead researchers if you’d like to contact them directly [provide the contact details for BK, Keneilwe or Luke as required]. If you have any other concerns I can also give you the contact details for the London School of Hygiene and Tropical Medicine Research Governance and Integrity Office.

Do you have any questions?

Are you happy to begin the interview?

##### Consent for the telephone survey

For the telephone survey, we will call potential participants and provide information about the purpose and risks of the telephone interview using an appropriate version of the Kenyan script shown below. Potential participants will have the opportunity to discuss the study and ask questions.

Good morning/afternoon

My name is … and I’m calling from the Vision Impact Project eye screening programme. We saw you a few weeks ago and referred you to the local clinic, but we did not see you on your appointed day.

In fact, half of all people who were referred did not attend. We have sought feedback on ways we could improve our service, and I wanted to ask you which of the ideas we have stand the best chance of helping people like you to access care. It should take approximately 15 minutes of your time.

If you are happy to proceed, I need to tell you a bit more about the survey. I will then double-check that you are still happy to proceed.

I will ask you about a set of potential changes that we are thinking about making. I will ask you to rate each one in terms of how likely you think it is to make a difference at helping people access our clinics.

Your responses will help us to shape and improve our services for others, but there are no direct benefits to you for taking part. Thinking about the issues that prevented you from getting care may be distressing to you. If you face any discomfort because of the questions asked, you can skip any question or ask to end the call whenever you choose.

If you don’t want to take part, that’s ok. You can drop out of the survey at any point. Your decision will not affect your health care or your future relations with the Vision Impact Project in any way.

Your anonymised answers will be combined with those from other people and kept safe and secure on password-protected computers in Nairobi and London. None of the data will be used for commercial use. We will publish our findings in a research journal and in a public repository so that other researchers can learn from what we find. You personal information will not be included in our findings and there is no way that you can be identified from any of the reports that we will produce.

If you have any questions, you can ask me now, or I can put you in contact with the study coordinator - Sarah Karanja from Kenya Medical Research Centre. If you have any questions about your rights as a research participant, I can connect you with the Kenya Medical Research Centre Ethics team who approved this survey.

Does that all make sense? Do you have any questions for me?

Are you happy for me to start?

##### Consent for participation in the workshop

All participants will be participating in the workshop as a routine part of their duties in connection with the respective eye programme. As such, consent is not required. The only output from this workshop will be the intervention(s) that will be implemented and evaluated using RCTs.

### Risks and strategies to mitigate

The risks to participants from the interviews, survey and focus group discussion are low and there are no physical risks. Dwelling on the issues that prevented attendance may cause psychological distress. Data collectors will be trained to supportively manage mild levels of distress and will signpost participants to other sources of support if participants become moderately or severely distressed.

Any issues, complaints or concerns will be reported to the principal investigators. Participants will be provided with their email addresses and office phone numbers. Participants will also be given the number of the local field coordinator for operational queries, and the LSHTM Research Governance and Integrity Office contact details for any other concerns about the conduct of the study.

We will compensate telephone interviewees for their time with an airtime voucher worth 100 BWP/500 KES/800 NPR (approximately £5). The voucher will be sent via SMS to telephone interviewees. Given the lower time and cognitive burden, survey responders will not be offered reimbursement, nor will workshop participants, as quality improvement is a core part of their role.

All data collected will be encrypted and stored on secure servers protected with strong authentication controls including two-factor authentication. All data will be processed and safeguarded in compliance with the EU and UK’s General Data Protection Regulation, as well as local regulation. Data will be anonymised and kept confidential. After 7 years, all study data will be destroyed. We have developed a robust Data Management Plan (online supplemental appendix 1).

### Ethics

This study has been approved by the ethics committee at the London School of Hygiene and Tropical Medicine (reference: 28415), the University of Botswana (reference: HPRD: 6/14/1), the Scientific Committee at Dr Schroff’s Charity Eye Hospital (reference: IRB/2023/JUN/161), the Kenya Medical Research Institute Scientific and Ethics Review Unit (reference: KEMRI/RES/7/3/1) and the Nepal Health Research Council (reference: 158).

### Dissemination

Our findings will be shared with lay representatives, community advisory board members, local and national programme funders and implementing partners, Peek Vision and national eye care policymakers. No participant names or identifiable information will be used. The study findings will also be disseminated during quarterly review meetings with implementing partners, community workers and representatives from the county health management committee and bi-annual partner meetings. We will also present our findings at national, regional and/or international conferences.

## Discussion

The series of elicitation elements in this study will produce a list of barriers to accessing eye health services, as perceived by patients or their proxies, as well as insight into what service modifications may be most useful for overcoming these barriers. The survey and workshop will refine this list, identifying those service modifications that are deemed to be most impactful by a representative sample of non-attenders, as well as offering the optimal balance of impact, cost and risk by programme managers. We will test this approach in four different settings, and aim to apply it for conditions beyond eye care in the future.

While our analytic framework is grounded in the literature, the obviation of transcription and dual coding by highly trained qualitative researchers clearly limits the reliability of the interview findings. We have deliberately sought to develop a method that can be deployed in low-resource settings where there are not necessarily qualitative researchers available and time is at a premium. Previous work has shown that rapid qualitative methods led by less-experienced research assistants are able to generate valid findings when the subject matter is not overly complex. We feel that seeking a list of potential barriers and solutions meets these criteria.

The highest-ranked potential service modifications will be presented to local and regional policymakers and stakeholders to garner their views on which should be prioritised for implementation, based on their likely impact, feasibility, cost and potential risks. Stakeholders include community advisory board representatives in each setting. By having community members assist with analysis and interpretation of study findings, this design provides a participatory approach to the selection of interventions and health service modifications that will be tested in subsequent work. Those responsible for funding and implementing the modifications will also play a role in reviewing data and selecting the most appropriate interventions to test.

Improvements in access to health services and health equity are the key component of this study, as we seek to focus on the needs of the most marginalised groups of non-attenders. We aim to refine and apply these methods to address other areas blighted by inequitable and low access.

### Limitations

Despite the fact that phone penetration is high in the countries we are working in, not everyone has their own phone and it is also likely that members of the most disadvantaged groups will be the least likely to respond to our telephone interviews and surveys, as well as being the least likely to attend services. It is possible that those with access to phones have different opinions on barriers and interventions and this could bias the results. In terms of alternatives, postal surveys are problematic for a range of other reasons including the lack of addresses, poor reliability of the postal service and issues with loss of data. In-person surveys would be the most robust way of ensuring that every voice is heard, but we do not have the time or resources given the national scale of the programmes.

### Study status

At the time of manuscript submission, we have piloted the approach in Kenya and obtained ethical approval for every country. We have not started recruitment or data collection in Botswana, India or Nepal.

### Study coordination centre

London School of Hygiene & Tropical Medicine. This trial will adhere to the principles outlined in the International Conference on Harmonisation Good Clinical Practice guidelines, protocol and all applicable local regulations.

## supplementary material

10.1136/bmjopen-2023-082975 online supplemental file 1
