# Supplementary material for: Identifying barriers and potential solutions to improve equitable access to community eye services: an exploratory sequential mixed methods study protocol
Source: BMJ Open. 2025 Jan 23;15(1):e082975. doi: 10.1136/bmjopen-2023-082975 (PMC11758701; doi:10.1136/bmjopen-2023-082975)
Supplement: Supplementary file 2 [file bmjopen-2023-082975.reviewer_comments.pdf]

PEER REVIEW HISTORY

BMJ Open publishes all reviews undertaken for accepted manuscripts. Reviewers are asked to complete a checklist review form and are provided with free text boxes to elaborate on their assessment. These free text comments are reproduced below.

ARTICLE DETAILS

Title (Provisional)

Identifying barriers and potential solutions to improve equitable access to community eye services: a rapid exploratory sequential mixed methods study protocol

Authors

Allen, Luke Nelson; Karanja , Sarah; Gichangi, Michael; Mishra, Sailesh; Sabherwal, Shalinder; Motlhatlhedi, Keneilwe; Nkomazana, Oathokwa; Macleod, David; Kim, Min; Ramke, Jacqueline; Ratshaa, Bakgaki; Tlhajoane, Malebogo; Ho-Foster, Ari; Tlhakanelo, John; Bolster, Nigel; Roshan, Abhishek; Javed, Mohd; Burton, Matthew J; Bastawrous, Andrew

---

VERSION 1 - REVIEW

**Reviewer**	**1**
**Name**	**Codina, Charlotte**
**Affiliation**	**The University of Sheffield, Ophthalmology & Orthoptics**
**Date**	**12-Feb-2024**
**COI**	**I do not have any competing interests to declare.**

The aims and ambitions of this research proposal are important and worthy. Increasing access to eye services is a vital global health priority. The large screening Peek Vision programmes and systems offer a unique opportunity to capture views on eye services, accessing these and barriers to this in Botswana, India, Kenya and Nepal, with the potential to improve access to key interventions such as glasses and cataract surgery. Improving the uptake of these in developing nations, would boost vision eye health globally, therefore the project is a worthy endeavour. Reviewing the protocol has raised questions, which I ask to maximise the meaningfulness of the study's output.

General questions

Why have these countries in particular been chosen for this study? It appears that tens of countries use the PEEK app, therefore why this grouping? The countries are quite disparate in many ways - geographically, culturally, socially, epidemiologically.

Abstract

Introduction: 'certain groups' is rather vague?

'Rapid yet robust mixed methods' is again, somewhat vague?

Ethics and dissemination:

Would be helpful to know which boards specifically have approved this study

Strengths and limitations

Line 24 'withing' should be 'within'

The 'approach' developed and reported in lines 8-14: has it been validated?

Line 26: Will the actionable and policy changes be at national level, regional level, or international level?

Page 9, line 47: It would be helpful to know how these 6 pieces of literature were identified and what criteria were used to conclude that they are 'well-conducted'.

Objectives

The first objective to conduct interviews with people from 'left behind' groups is a worthy aim, but is potentially too broad to be useful. For example, a 'left behind' group could be a group of sex workers, transgender, unemployed, cancer survivors, domestic abuse survivors, ex-convicts etc.. Each of these groups from each of the different countries will have very different reasons for not accessing services. Even within these groups the reasons are likely to be varied. How will meaningful conclusions be able to be drawn from combining 'left behind groups' in this way?

I am unsure what a representative sample would look like from so broad a group?

Within these countries and even within the 'left behind' groups that will be identified, there will be significant differences between regions for example, rural and urban reasons for non-attendance are likely to be quite different.

Methodology

Presuming 'each location' relates to each country and having worked in eyecare myself in Africa and Nepal, I cannot see how this question can be answered on a national level. E.g. those in capital cities can reach healthcare more easily than for those for whom it is a two-day walk away.

Will children and young people's voices be heard in this study? This is not clear throughout, but it is noted that access has been found to be particularly low in the Kenyan pilot, in 'youn people'. Are there any considerations around this in terms of familial hierarchies in some settings and cultures e.g. inappropriate for a mother to consent for a 15 year old son when he might be considered the head of the family?

Page 31, line 43: If all interviews will be conducted in the participant's home language, who will do the translation and how will this be checked? How will you ensure that the person

conducting the interview, is a psychologically safe, approachable, confidentially bound and sensitive to potentially very sensitive information? How will you handle participants who find the experience of sharing barriers to accessing healthcare emotionally distressing? (e.g. 'my husband won't let me', 'my family believe glasses will make our daughters less marriageable', 'we seek the local healer instead' etc.)

The questions to be asked on the telephone are good, but will they go deep enough to uncover some of the difficult truths of the matter, without a face to face arm, such as a focus group where some of these questions could be more deeply explored? I'm not sure that the telephone interview will create enough of a trusting, collaborative alliance, to uncover the most difficult aspects of barriers to vision care.

The research team may wish to consider a face to face 'arm' to the study to capture the voices of those most affected by poverty - both digital and physical. This would allow the voice of the most disadvantaged, most 'left behind' to be additionally heard and would create room for a greater development of the trust equation, to uncover deeper barriers embedded within these regions and settings which limit access to healthcare.

**Reviewer**	**2**
**Name**	**Ezinne, Ngozi**
**Affiliation**	**The University of the West Indies**
**Date**	**07-Jun-2024**
**COI**	**I have no competing interest**

The protocol seems to be too long and not well organized.

The authors failed to introduce the topic well and provide justification for the study need.

The manuscript lacked information on the detailed steps that will be taken for data collection.

The study was not reader friendly and had a lot of irrelevant information for a protocol

---

**Reviewer**	**3**
**Name**	**Bernhisel, ashlie**
**Affiliation**	**University of Utah Health**
**Date**	**24-Jun-2024**
**COI**	**None**

Excellent work. One question that does not affect acceptance: In terms of promoting participatory research in the local community, this proposal did not address how the local community was involved in generating the research question. I assume local stakeholders were involved in the process?

VERSION 1 - AUTHOR RESPONSE

**Point for Clarification**		**Response**
**Reviewer 1**		
1.	The aims and ambitions of this research proposal are important and worthy. Increasing access to eye services is a vital global health priority. The large screening Peek Vision programmes and systems offer a unique opportunity to capture views on eye services, accessing these and barriers to this in Botswana, India, Kenya and Nepal, with the potential to improve access to key interventions such as glasses and cataract surgery. Improving the uptake of these in developing nations, would boost vision eye health globally, therefore the project is a worthy endeavour. Reviewing the protocol has raised questions, which I ask to maximise the meaningfulness of the study's output.	No response required.
2.	Why have these countries in particular been chosen for this study? It appears that tens of countries use the PEEK app, therefore why this grouping? The countries are quite disparate in many ways - geographically, culturally, socially, epidemiologically.	We have added the following explanation to the text:<br><br>*We selected these four countries because they represented different populations and screening programme types; had screening programme commencement dates that aligned with our project timeline; and were overseen by managers with pre-existing academic collaborative relationships with the LSHTM team.*
3.	Abstract<br><br>Introduction: 'certain groups' is rather vague?	We can't be much more specific, as the groups that face the highest barriers to care will vary by location and setting. For instance, previous work by our team has found that older female widows are the least likely to access eye care in Nigeria, but it was younger men that were the least likely to access care in Kenya. To try and improve clarity we have changed the wording to:

		Access to care varies by sociodemographic group, with some groups facing higher barriers to care than others.
4.	'Rapid yet robust mixed methods' is again, somewhat vague?	We don't have the wordcount in the abstract to unpack this much further. We have added the following clarifying text:<br><br>*; delivering non-tokenistic findings within a matter of weeks.*
5.	Ethics and dissemination:<br>Would be helpful to know which boards specifically have approved this study	This study has been approved by the ethics committee at the London School of Hygiene and Tropical Medicine and the ethics boards within each of the four study countries. The following statement has been added to page 31 of the manuscript for further information.<br><br>*Ethics*<br>*This study has been approved by the ethics committee at the London School of Hygiene and Tropical Medicine (ref: 28415), the University of Botswana (ref: HPRD: 6/14/1), the Scientific Committee at Dr Schroff's Charity Eye Hospital (ref: IRB/2023/JUN/161), the Kenya Medical Research Institute Scientific and Ethics Review Unit (SERU) (ref: KEMRI/RES/7/3/1), and the Nepal Health Research Council (ref: 158).*
6.	Strengths and limitations<br>Line 24 'withing' should be 'within'	This error has been corrected.
7.	The 'approach' developed and reported in lines 8-14: has it been validated?	This paper is the protocol for the novel approach. As such it has not been tested or validated yet. The following underlined text has been added to the end of line 14:<br><br>We have developed a bespoke rapid qualitative approach that is designed to deliver rich and robust data with speed and relatively low costs. Our approach is based on a prior scoping review of rapid methods. <u>It is yet to be tested and validated</u>.
8.	Line 26: Will the actionable and policy changes be at national level, regional level, or international level?	Given that the approach is embedded within real-world programmes, it depends on the scale of the underlying programme.<br><br>Some of the programmes are run regionally (e.g. the Indian programme operating in Uttar Pradesh), some nationally (e.g. Botswana's

		national Pono Yame programme), while some programmes are run by local government (e.g. Kenya's Vision Impact Programme), others by the national governments or NGOs (e.g. Nepal's screening programmr). Any policy changes will be determined by the specific context in each country and the nature of the eye screening programme.
9.	Page 9, line 47: It would be helpful to know how these 6 pieces of literature were identified and what criteria were used to conclude that they are 'well-conducted'.	We have removed the opening sentence in question. The paragraph now simply presents six papers that have previously used qualitative methods to examine access to eye care, without making definitive claims about their quality or the exhaustiveness of our search.
10.	Objectives: The first objective to conduct interviews with people from 'left behind' groups is a worthy aim, but is potentially too broad to be useful. For example, a 'left behind' group could be a group of sex workers, transgender, unemployed, cancer survivors, domestic abuse survivors, ex-convicts etc.. Each of these groups from each of the different countries will have very different reasons for not accessing services. Even within these groups the reasons are likely to be varied. How will meaningful conclusions be able to be drawn from combining 'left behind groups' in this way?	The method is designed to focus on whatever group has been found to face the lowest overall odds of attendance based on a preceding equity analysis. We have added references to the protocol for this preceding work and some published real-world examples.<br><br>The reviewer is absolutely correct that the left behind group sis likely to be different in every programme, and their reasons are likely to be varied.<br><br>We are not attempting to draw conclusions across programmes. We are presenting an approach to be used in each individual country/programme to deliver unique findings that can be applied to each unique programme and population. We state that:<br><br>*We intend to test this approach in four different eye screening programmes running in Botswana, India, Kenya, and Nepal and then apply the findings within the same services with the ultimate aim of improving equitable access to care. Findings from one programme will not be applied to the others, although learning will be shared across sites….*<br><br>*We will use a mixed methods approach; starting with qualitative methods to explore non-attenders' perceptions of the barriers and potential solutions in each setting. We will use the identified themes to develop a unique, user-derived list of potential service modifications within each screening programme.*

		Our use of the representative ranking survey enables us to take findings from the interviews and end up with a ranked list of solutions that apply across the entire population within each programme.
11.	Objectives: I am unsure what a representative sample would look like from so broad a group?	We use standard statistical sampling techniques. Further detail is provided in the 'Survey' section of the Methods:<br><br>We will use a 95% confidence interval, a 5% margin of error, and a conservative assumption that the total population size is 1 million people (with the same characteristics as the most marginalised group). This renders a sample size of 384.
12.	Objectives: Within these countries and even within the 'left behind' groups that will be identified, there will be significant differences between regions for example, rural and urban reasons for non-attendance are likely to be quite different.	We do not seek to make comparisons across countries, or even across programmes within countries.<br><br>The unit of analysis is the population of each programme. Once an equity analysis has identified which sociodemographic characteristics are most strongly associated with non-attendance, we perform a set of interviews with people from this group to identify their perceptions of barriers and potential solutions. We have removed mention of the four countries from the title to stress that we are not aiming to conduct cross-country comparisons.<br><br>We continue interviewing until reaching saturation in each programme. We also aim to include a maximum variation sample
13.	Methodology: Presuming 'each location' relates to each country and having worked in eyecare myself in Africa and Nepal, I cannot see how this question can be answered on a national level. E.g. those in capital cities can reach healthcare more easily than for those for whom it is a two-day walk away.	Again, our original manuscript was not clear enough and we have added new wording, in this instance to explain that the approach is embedded within individual screening programmes:<br><br>…what are the main barriers to accessing eye services in each location (<u>i.e. each individual screening programme in each of the four countries</u>)…
14.	Methodology: Will children and young people's voices be heard in this study? This is not clear throughout, but it is noted that access has been found to be particularly low in the Kenyan pilot, in 'youn people'. Are there any considerations	The programmes in Kenya, Nepal, and India all focus on adults (>18 years). The Botswana programme is focused on school children (5-18 years), however for this first iteration we are only interviewing parents/guardians. This is

	around this in terms of familial hierarchies in some settings and cultures e.g. inappropriate for a mother to consent for a 15 year old son when he might be considered the head of the family?	mainly for reasons of feasibility (e.g. children often stay at boarding schools and don't have phones we can reach them on for the interviews and surveys). Once we have proof of concept, we will aim to include younger voices.
15.	Page 31, line 43: If all interviews will be conducted in the participant's home language, who will do the translation and how will this be checked? How will you ensure that the person conducting the interview, is a psychologically safe, approachable, confidentially bound and sensitive to potentially very sensitive information? How will you handle participants who find the experience of sharing barriers to accessing healthcare emotionally distressing? (e.g. 'my husband won't let me', 'my family believe glasses will make our daughters less marriageable', 'we seek the local healer instead' etc.)	In each setting we use bi/multilingual research assistants to conduct the interviews. They all receive additional training in the ethical and sensitive conduct of qualitative research and are evaluated performing simulated interviews and pilot work before starting work in the field. They are supervised by experienced qualitative researchers and have a daily debrief session to discuss any challenging issues that arise. Should a participant become distressed, the interviewer follows a protocol that has been reviewed and approved by the local ethics committee. Note that p29 already includes the following information:<br><br>*Dwelling on the issues that prevented attendance may cause psychological distress. Data collectors will be trained to supportively manage mild levels of distress and will signpost participants to other sources of support if participants become moderately or severely distressed.*<br>*Any issues, complaints or concerns will be reported to the principal investigators. Participants will be provided with their email addresses and office phone numbers. Participants will also be given the number of the local field coordinator for operational queries, and the LSHTM RGIO contact details for any other concerns about the conduct of the study.*<br><br>Research assistants enter quotes into the analytic matrix under the supervision of a multilingual senior researcher who performs quality checks and reviews a sample of the original audio files.<br><br>We have added new text to add this detail.
16.	The questions to be asked on the telephone are good, but will they go deep enough to uncover some of the difficult truths of the matter, without a face to face arm, such as a focus group where some of these questions could be more deeply explored? I'm not sure that the telephone interview will create enough of a trusting, collaborative alliance, to uncover the	We have the same questions, and have received ethical approval to conduct a nested face-to-face vs telephone comparison. This study will be reported separately.<br><br>*Allen, L. N., Karanja, S., Tlhajoane, M., Tlhakanelo, J., Macleod, D., & Bastawrous, A. (2024). A Protocol for the Comparison of*

	most difficult aspects of barriers to vision care. The research team may wish to consider a face to face 'arm' to the study to capture the voices of those most affected by poverty - both digital and physical. This would allow the voice of the most disadvantaged, most 'left behind' to be additionally heard and would create room for a greater development of the trust equation, to uncover deeper barriers embedded within these regions and settings which limit access to healthcare.	*Telephone and In-Person Interview Modalities: Duration, Richness, and Costs in the Context of Exploring Determinants of Equitable Access to Community Health Services in Meru, Kenya. International Journal of Qualitative Methods, 23.*
**Reviewer 2**		
18.	The protocol seems to be too long and not well organized.	As this is a mixed methods project, containing a qualitative study and a fully-fledged survey, the protocol is longer than a standard single-method document. We wanted to thoroughly and robustly justify exactly what we were doing and why, following the principles of open science.<br><br>The paper structure follows those set out in the journal's instructions to authors, but have moved the context section from the intro to the methods which helps with the flow.
19.	The authors failed to introduce the topic well and provide justification for the study need.	We were aware that the methods description was long, and so tried to keep the opening paragraphs as short as possible. We have now added additional context to the start of the paper and explicitly laid out why the study is needed.
20.	The manuscript lacked information on the detailed steps that will be taken for data collection.	We have added additional text to explain the detailed steps for data collection throughout the methods to make the steps more explicit.
21.	The study was not reader friendly and had a lot of irrelevant information for a protocol	This manuscript is the foundation document for quite a complex and innovative mixed-methods study. As well as providing the standard step-by-step instructions on how to go about gathering and collecting data, we felt that it was important to lay out the more fundamental background on why this new rapid approach is needed, how it fits in with- and builds on existing approaches in this field, and what the theoretical grounding is. We envisage receiving lots of questions from qualitative researchers and want to be able to point them to this

		background document that spells out exactly what we did and why.
**Reviewer 3**		
22.	Excellent work.	No response required.
23.	One question that does not affect acceptance: In terms of promoting participatory research in the local community, this proposal did not address how the local community was involved in generating the research question. I assume local stakeholders were involved in the process?	Local stakeholders co-designed the study and are co-authors. The study design itself centres around the voice and perspective of left behind groups and places their suggestions at the heart of efforts to modify and improve services.

VERSION 2 - REVIEW

Reviewer **1**

Name **Codina, Charlotte**

Affiliation **The University of Sheffield, Ophthalmology & Orthoptics**

Date **08-Nov-2024**

COI

Thank you for addressing the comments so thoroughly. The paper is much improved and I have no further comments to add.
